# Predictors' Factors of Nutritional Status of Male Chronic Obstructive Pulmonary Disease Patients

**DOI:** 10.5402/2012/782626

**Published:** 2012-11-14

**Authors:** Elham Pirabbasi, Mahin Najafiyan, Maria Cheraghi, Suzana Shahar, Zahara Abdul Manaf, Norfadilah Rajab, Roslina Abdul Manap

**Affiliations:** ^1^Dietetic Programme, Department of Nursing and Midwifery, Abadan Faculty of Medical Health Sciences, Ahwaz Jundishapur University of Medical Sciences, Ahwaz 6135715794, Iran; ^2^Dietetic Programme, Center for Health Care Sciences, Faculty of Health Sciences, National University of Malaysia, 50300 Kuala Lumpur, Malaysia; ^3^Department of Obstetrics & Gynecology Medical School, Ahwaz Jundishapur University of Medical Sciences, Ahwaz 6135715794, Iran; ^4^Department of Public Health, Faculty of Health Sciences, Ahwaz Jundishapur University of Medical Sciences, Ahwaz 6135715794, Iran; ^5^Biomedical Programme, Faculty of Health Sciences, National University of Malaysia, 50300 Kuala Lumpur, Malaysia; ^6^Department of Medicine, Faculty of Medicine, National University of Malaysia, 56000 Cheras, Malaysia

## Abstract

Chronic obstructive pulmonary disease (COPD) is a systemic disease that leads to weight loss and muscle dysfunction resulting in an increase in mortality. This study aimed to determine the prevalence rate of malnutrition and nutritional status and also factors associated with nutritional status. A total of 149 subjects were involved in the cross-sectional study. The study was conducted at two medical centers in Kuala Lumpur, Malaysia. The results of the study showed that malnutrition was more prevalent (52.4%) in the subjects with severe stages of COPD as compared to mild and moderate COPD stages (26.2%) (*P* < 0.05). Fat-free mass depletion as assessed using fat-free mass index (FFMI) affected 41.9% of the subjects. Plasma vitamin A, peak expiratory flow (PEF), and handgrip were the predictors for body mass index (BMI) (*R* ^2^ = 0.190, *P* < 0.001). Plasma vitamin A and force expiratory volume in one second (FEV_1_) were the predictors of FFMI (*R* ^2^ = 0.082, *P* = 0.007). BMI was the predictor of respiratory factors, that is, FEV_1_% predicted (*R* ^2^ = 0.052, *P* = 0.011). It can be concluded that there is a need to identify malnourished COPD patients for an appropriate nutrition intervention.

## 1. Introduction

Chronic obstructive pulmonary disease (COPD) is a treatable and preventable disease. It is characterized by an airflow limitation which is not fully reversible [1]. COPD affects the lungs and produces significant systemic consequences such as weight loss and muscle dysfunction [2]. Weight loss and depletion of fat-free mass (FFM) may be observed in stable COPD patients, irrespective of the degree of airflow limitation. Weight loss and underweight are associated with an increased mortality risk. Weight loss and particularly muscle wasting contributes significantly to morbidity, disability, and handicap in COPD patients [1]. Skeletal muscle wasting is commonly present in patients with COPD and may also be present in patients with a stable weight [1]. Weight loss and loss of fat mass are primarily the result of a negative balance between dietary intake and energy expenditure, while muscle wasting is a consequence of an impaired balance between protein synthesis and protein breakdown. In advanced stages of COPD both energy balance and protein balance are disturbed. Therefore, nutritional therapy may only be effective if combined with exercise or other anabolic stimuli. Nutritional intervention should focus more on prevention and early treatment of weight loss to preserve energy balance [1]. 

Weight loss and being underweight are associated with decreased diffusing capacity and are observed more frequently in emphysematous patients than those with chronic bronchitis [3, 4]. A higher myofibrillar protein breakdown has been seen in cachexic COPD patients compared to noncachexic patients and healthy controls [5]. Furthermore, elevated activity induced and daily energy expenditure have been observed in ambulatory COPD patients [6]. As a consequence of this elevated energy metabolism, COPD patients who suffer from weight loss, and even some whose weight is stable, may lose weight despite an apparently normal energy intake. In addition, the symptoms of the disease and an enhanced systemic inflammatory response may affect appetite and dietary intake. Increased muscle protein breakdown is a key feature in muscle wasting. This process of cachexia can be considered the result of interplay of systemic factors, including systemic inflammation, oxidative stress, and growth factors that may synergize with local factors leading to protein imbalance [7]. The prevalence of malnutrition among patients with COPD ranges from 20 to 50%, depending on disease severity [8]. Recent study showed that prevalence of malnutrition among COPD patients in Malaysia was 12.7% [9]. Therefore, frequent nutritional assessment is needed to prevent of malnutrition among COPD patients particularly in those with severe COPD stages.

## 2. Methodology

### 2.1. Study Design and Subjects

A cross-sectional study was conducted to determine the nutritional status of male COPD patients. Factors associated with poor nutritional status were also determined. The study was carried out at outpatients' department (OPD) from the Medical Center of National University of Malaysia and Institute of Respiratory Medicine. The study started from July 2009 until January 2010. Inclusion criteria for the study were male diagnosed with COPD regardless to age, smoking status, and COPD stages. The exclusion criteria were inflammatory diseases such as rheumatoid arthritis, tuberculosis, and bronchitis. The study was approved by the National University of Malaysia Research Ethics Committee (UKM 1.5.3.5/244/SPP/NN-056-2009) and also was registered in an open access database trial on 20 January 2012 (ISRCTN18062761). Written consent has been obtained from each subject. A total of 149 subjects who were referred to Medical Center of National University of Malaysia and Institute of Respiratory Medicine were participated in this study. All patients diagnosed with COPD by the physician and who met the inclusion criteria were interviewed to participate. Out of 149 subjects, 52 subjects refused for blood drawing and 44 subjects refused to measure body composition which required fasting at least 8 hours prior to the procedure. Thus, 105 subjects completed body composition measurement and 149 subjects completed nutritional and respiratory functional assessment.

### 2.2. Sample Size Calculation and Sampling Method

The formula for sample size calculation was used from below formula [10]:

(1) $$\begin{array}{c} N\left(\text{subject}\right)=\frac{\left(z^{2}\right)pq}{d^{2}}. \end{array}$$

In this formula, the power of study was estimated by 90% and significant level of 0.05 ( = *d*), *z* is constant = 1.645 and *q* = 1 − *p*, *p* = was the prevalence of malnutrition among COPD patients in Malaysia, which is 12.7% (= 0.127) [9]. Consider

(2) $$\begin{array}{c} N=\frac{{\left(1.645\right)}^{2}\left(0.127\right)\left(1-\;0.127\right)}{{\left(0.05\right)}^{2}}=120,\\ N=120\times 25\%\left(\text{account}\;\;\text{for}\;\;\text{attrition}\right)=150. \end{array}$$

The convenience (nonprobability) sampling method was used. The subjects were the selected because they were the easiest to recruit for the study and research did not consider selecting subjects that are represented of the entire population. 

### 2.3. Measurements

Nutritional status was assessed according to anthropometry indicators including, weight, height, mid upper arm circumference (MUAC), triceps skin fold thickness (TSFT) waist circumference (WC) [11, 12], calf circumference (CC) [13], hip circumference (HC) [14] and body composition including, fat-free mass (FFM), fat mass (FM), and handgrip strength. Dietary intakes (one day recall and two days record) were also obtained. Subjects were asked to record all foods and drinks consumed for one weekday and one weekend using household measures. The food record was collected during next followup. The dietary intake was analysed using Nutritionist-Pro software. Body composition was measured using Maltron body fat analyzer (Maltron, B-F 900, England) for which the subject should be fast at least 2-3 hours and urinate 30 minutes prior to the procedure. The handgrip strength also measured using handgrip strength (GRIP-D, T.K.K 5401, Japan). Lung function was assessed using Spirometry equipment, that is, Spirobank G (AAA MIR, A23-04104545, Italy).

**Biochemical Measurement**

A total of 10 mL fasting venous blood was drawn for measurement of antioxidant status such as vitamin A, vitamin E, vitamin C, glutathione, and total antioxidant capacity.   In order to measure blood plasma vitamin A and E, Heparinized tube (BD Vacutainer lithium heparin 87 USP units, Ref 367886, USA) was used. Vitamin C (L-ascorbic acid, purity 99%), Vitamin E (±*α*-tocopherol, purity 95%), and vitamin A (*β*-carotene, purity 97%) used were obtained from Sigma Chemical Company (St. Louis, Mo, USA). The methanol, acetonitrile, and tetrahydrofuran used were obtained from Merck KGaA (Darmstadt, Germany). All solvents were of HPLC grade. Vitamin A (retinol) and E (*α*-tocopherol) in serum/plasma were measured by isocratic high performance liquid chromatography (HPLC) with detection at three different wavelengths. The Bio-Rad vitamin A/E serum was used as a calibrator for the Bio-Rad vitamin A/E by HPLC test (Ref 195–5869, GmbH, Germany), for calculating the concentrations of vitamins A or E in patient specimen. The reference range was revised values for the interpretation of serum or plasma retinol and *α*-tocopherol based on the trials done on 65 apparently healthy Malaysians with a mean age of 52.8 years (range 24 to 76 years) [15].

In order to measure blood plasma vitamin C, Heparinized tube (BD Vacutainer lithium heparin 87 USP units, Ref 367886, USA) was used. About 1.5 mL venous blood was required to obtain 0.5 mL plasma to measure vitamin C. The plasma was obtained after centrifuging to remove the clot and blood cells. The plasma was mixed with the oxalic acid preservative and wrapped with the aluminum foil to keep at −70°C until run. Vitamin C (L-ascorbic acid, purity: 99%) was obtained from Sigma Chemical Co. (St. Louis, Mo, USA). Methanol, acetonitrile, and tetrahydrofuran were obtained from Merck KGaA (Darmstadt, Germany). All solvents were HPLC grade. All the reagents were used without further purification. Deionized water, purified by Milli Q system (Millipore, Milford, MA, USA) was used throughout the procedure [16]. 

Total antioxidant capacity was measured by the ferric reducing antioxidant power (FRAP) assay [17]. In order to measure plasma glutathione, EDTA tube (BD Vacutainer K2 EDTA10.8 mg, Ref 367863, USA) was used. About 1 mL fasting venous blood was extracted to obtain 0.5 mL plasma for measuring plasma glutathione. The sample was stored at −20°C up to −70°C until analyzed. The Calbiochem GSH Assay Kit II (Cat. No. 354103) was used to determine the concentration of GSH in human plasma.

## 3. Statistical Analysis

Data were analyzed using Statistical Package for Social Sciences (SPSS) version 16. A Kolmogorov-Smirnov test was performed prior to statistical analysis in order to examine the normality of the variables. In order to find the relationship between parametric and nonparametric variables, Pearson Correlation and Spearman Coefficient Correlation were applied, respectively. Independent sample *t*-test and Mann-Whitney *U* test for parametric and nonparametric variables to compare mean of two groups were applied. In addition, to compare means of more than two groups with one normally continuous variable, one-way ANOVA test and for nonparametric variable Kruskal-Wallis test were performed. In order to find out the association between 2 grouping variable (2 × 2 table), chi-squared test was performed. The predictors for nutritional status and respiratory function were determined using multiple linear regression to find the best model. The entire statistical tests the *P* value was significant at the level of 0.05.

## 4. Results

Nutritional status of the subjects based on BMI showed that about 45% of the subjects had normal body weight, followed by overweight/obese (37.6%) and underweight (17.4%). About 47.6% of the subjects aged 60 years and above were normal weight or over/obese (38.1%) but 25% of younger age group were underweight (Table 1). Mean of BMI of the subjects was 23.5 ± 4.8 kg/m^2^. According to the equation by VanItallie et al. [18], 41.9% of respondents had depletion of fat-free mass index (Table 1). Assessment of body fat percentile of the subjects based on TSFT showed that 94.6% had average or above average body fat. A total of 24.2% of the subjects had peripheral muscle wasting as assessed using MUAC. Handgrip strength results showed that 77.9% of patients had protein malnutrition. Prevalence of abdominal obesity (defined as waist circumference > 90 cm for Asian men) was 43.6% (Table 1). About 23.8% of the subjects had cachexia and 18.1% had muscle atrophy (Table 2). Malnutrition was more prevalent (52.4%) in the subjects with stages III and IV as compared to COPD stages I and II (26.2%) (*P* < 0.05) (Table 3). Assessment of food intake showed that calorie intake of the subjects in both younger and older age groups were below the Malaysian RNI [19], as shown in Table 4. Similar trend was noted for protein, vitamin E, and vitamin C intake. More than 65% of the subjects had an inadequate intake of energy, protein, vitamin C and E. Vitamin C intake was more deficient in older age group (95.2%) as compared to younger age group (90.9%) (*P* < 0.05) (Table 4).

### 4.1. Predictors of Nutritional Status

In order to find out the predictors for nutritional status, that is, BMI and FFMI, a univariate analysis was performed to find out the significant association (Table was not shown). Variables found to be significant in the univariate analysis were entered into multiple linear regression analysis to determine the best predictors. The highest adjusted *R* square was the best model predictors. Using univariate analysis, it was found that income, handgrip strength, vitamin E intake, FEV_1_% predicted, FEV_1,_ PEF, and PEF% predicted were associated with BMI. Handgrip strength, FEV_1_% predicted, FEV_1, _PEF and PEF% predicted were associated with FFMI. Multiple regression analysis indicated that respiratory function as assessed using PEF, plasma vitamin A, and handgrip strength were significant predictors of BMI (*R* ^2^ = 0.190, *P* < 0.001) as shown in Table 5. The predictors for FFMI were FEV_1_ and plasma vitamin A (*R* ^2^ = 0.081, *P* < 0.05) (Table 5).

## 5. Discussion

The study evaluated the nutritional status among COPD patients. Nutritional status of the subjects based on fat-free mass index (FFMI) showed that about 42% of the subjects had depletion of fat-free mass and muscle wasting. Depletion of fat-free mass has been reported in 20% of COPD outpatients [3] and 35% of those eligible for pulmonary rehabilitation [20]. Another recent study among COPD patients reported that the prevalence of fat-free mass depletion was about 30% [21]. 

In this study according to FFMI and BMI category of COPD subjects, 23.8% had cachexia and 18.1% had muscle atrophy. Malnutrition was associated with severe COPD which observed in 52.4% of patients with stages III and IV. Several studies also have been showed that malnutrition affected 50% of COPD patients with advance stage of disease [22, 23]. In this study, in addition to BMI, other anthropometry parameters, that is, mid upper arm circumference (MUAC), calf circumference (CC), and triceps skin fold thickness (TSFT) were associated with severe stage of COPD which indicated malnutrition in those patients. Similar result was found in a study conducted by Yang et al. [24] which reported that MUAC, TSFT, and MAMC were significantly lower in malnourished COPD. 

Besides malnutrition, a substantial percentage of obesity among COPD subjects with an enhanced BMI was also found. In this study, the prevalence of overweight/obesity of 37.6% was higher than those reported among COPD patients in US (27%) and Sweden (19%) [25, 26]. It is important to recognize that muscle mass may be reduced despite a normal BMI [20]. Increased in BMI does not protect against fat-free mass depletion in COPD, since there is a preferential loss of muscle tissue in this disease [20]. Peripheral muscle weakness as assessed by handgrip strength in this study showed that 77.9% of the subjects had protein malnutrition. This study found that energy, protein, vitamin E, and vitamin C intake of the subjects was below Malaysian RNI [19], especially in the older age group. This finding was also similar to other studies among Malaysian elderly [27]. A more recent study among the Malaysian elderly also described similar results of low energy intake as well as inadequate thiamin, riboflavin, and calcium intake [28]. 

In the current study, results showed that the predictors for nutritional status, that is, BMI were PEF, plasma vitamin A, and handgrip strength. The predictors for FFMI were FEV_1_ and plasma vitamin A. Based on the authors' knowledge no study showed the predictors of nutritional status among COPD patients, while other study by Landbo et al. [29] in Denmark, showed that low BMI was predictive of a poor prognosis (i.e., higher mortality), with relative risks in underweight subjects as compared with normal weight subjects of 1.64 (95% CI: 1.20 to 2.23) in men and 1.42 (95% CI: 1.07 to 1.89) in women. However, the association between BMI and survival differed significantly with different stages of COPD. Similar results were found for COPD-related deaths, with the strongest associations found in severe COPD (Relative risk for low versus high BMI: 7.11 (95% CI: 2.97 to 17.050) [29].

Based on our findings, nutritional status is an important part of COPD treatment, and there are criteria recommended for nutritional assessment and interventions among patients with COPD. A study by Odencrants et al. [30] investigated showed and how registered nurses (RNs) in primary care described nutritional assessment practices and interventions in COPD patients with impaired nutritional status. The RNs reported that their assessment of nutritional status was based largely on intuition. It seemed that RNs used intuition because of a lack of knowledge of systematic methods of nutritional assessment. The findings also indicated that the RNs attempted to build a relationship of trust with the patients rather than provide early information on sensitive topics (e.g., nutritional information) [30]. Therefore, further study with a comprehensive nutritional assessment should be investigated with close collaboration of physicians, nurses, and dietitians to prevent nutritional impairment among COPD patients.

## 6. Conclusion

From these pieces of evidence regarding nutritional status of COPD patients and its association between weight loss and severity of disease, it could be concluded that early detection of weight loss which is leading to malnutrition is important to prevent severity of disease. There is a need for frequent nutritional assessment or possibility of nutrition intervention to prevent severity of COPD stages. The patients should be referred to nutrition department for nutrition counseling. This programme needs to a professional healthcare team consisting of physicians, nurses, and dietitians. 

## 7. Limitation of the Study

The study was carried out to evaluate nutritional status among COPD patients only at two medical centers in Kuala Lumpur. According to a pretest study, data from patients' files record showed that prevalence of COPD among females was very low. Therefore, only male subjects were enrolled in this study. In the future, a multicenter study is recommended among both male and female subjects to assess and compare further nutritional requirement. 

## Figures and Tables

**Table 1:** Nutritional status based on anthropometry Measurement and handgrip strength of COPD subjects according to age group (*n* = 149).

Variable	Classification	35–59 yrs (*n* = 44)		≥60 yrs (*n* = 105)		Total (*n* = 149)		*P*
		*N*	%	*N*	%	*n*	%	
Body mass index (kg/m^2^)	Under weight (BMI < 18.5 )	11	25	15	14.3	26	17.4	—
	Normal (18.5 < BMI < 24.9)	17	38.6	50	47.6	67	45.0	
	Overweight/obese (BMI > 25)	16	36.4	40	38.1	56	37.6	
Triceps skin fold thickness (mm)	Lean/below average (TSFT < 15th)	1	2.3	7	6.7	8	5.4	—
	Average (15th < TSFT < 75th)	27	61.4	57	54.3	84	56.4	
	Above average/obese (TSFT > 75th)	16	36.4	41	39.0	57	38.3	
Mid-upper arm circumference (cm)	Under weight (MUAC < 23.5)	11	25	25	23.8	36	24.2	—
	Normal (23.5 < MUAC < 32)	28	63.6	78	74.3	106	71.1	
	Obese (MUAC > 32)	5	11.4	2	1.9	7	4.7	
Waist circumference (cm)	Normal (WC < 90)	28	63.6	56	53.3	84	56.4	0.022^a^
	Abdominal obesity (WC ≥ 90)	16	36.4	49	46.7	65	43.6	
Fat-Free Mass Index (kg/m^2^) (*n* = 105)	<16 (kg/m^2^) depletion of FFM	16	50.0	28	38.4	44	41.9	0.266
	≥16 (kg/m^2^) Normal	16	50.0	45	61.6	61	58.1	
Handgrip (kg) malnutrition	Handgrip < 34 kg, age < 69 year	32	72.7	82	78.1	114	76.5	—
	Handgrip < 27.5 kg, age 70–79 year	0	0.0	1	1.0	1	0.7	
	Handgrip ≤ 19 kg, age ≥ 80 year	0	0.0	1	1.0	1	0.7	
Normal	Handgrip > 34 kg, age < 69 year	12	27.3	3	2.9	15	10.1	
	Handgrip < 27.5 kg, age 70–79 year	0	0.0	16	15.2	16	10.7	
	Handgrip ≤ 19 kg, age ≥ 80 year	0	0.0	2	1.9	2	1.3	

**Table 2:** Prevalence of malnutrition according to FFMI and BMI among COPD subjects (expressed as number and %).

Body composition classification		Number (*n* = 105)	Percentage (%)
Malnutrition			
Cachexia	FFMI < 16 kg/m^2^	25	23.8
	BMI < 21 kg/m^2^		
Muscle Atrophy	FFMI < 16 kg/m^2^	19	18.1
	BMI ≥ 21 kg/m^2^		
Normal			
Starvation	FFMI ≥ 16 kg/m^2^	6	5.7
	BMI < 21 kg/m^2^		
No changes	FFMI ≥ 16 kg/m^2^	55	52.4
	BMI ≥ 21 kg/m^2^		

**Table 3:** Prevalence of malnutrition of subjects according to FFMI and COPD staging (expressed as number and %).

Variable		FFMI (*n* = 105)				*P*
		<16 kg/m^2^		≥16 kg/m^2^		
		*n*	%	*n*	%	
COPD	Stage I, II	11	26.2	31	73.8	0.008
	Stage III, IV	33	52.4	30	47.6	

*P* < 0.05 significant using Pearson chi-square test.

**Table 4:** Percentage of food intake according to Malaysian RNI among age group (*n* = 149).

Variable		30–50 years (*n* = 44)		≥60 years (*n* = 105)		Total (*n* = 149)	
		*N*	%	*n*	%	*n*	%
Energy (kcal/day)	< RNI	44	100	95	90.5	139	93.3
	≥ RNI	0.0	0.0	10	9.5	10	6.7
Protein (gr/day)	< RNI	30	68.2	71	67.6	101	67.8
	≥ RNI	14	31.8	34	32.4	48	32.2
Vitamin A (RE/day)	< RNI	13	29.5	32	30.5	45	30.2
	≥ RNI	31	70.5	73	69.5	104	69.8
Vitamin E (mg/day)	< RNI	44	100	105	100	149	100.0
	≥ RNI	0.0	0.0	0.0	0.0	0.0	0.0
Vitamin C (mg/day)	< RNI	40*	90.9	100	95.2	140	94.0
	≥ RNI	4	9.1	5	4.8	9	6.0

*Significant at the level of *P* < 0.05.

**Table 5:** Multiple regression model predicting subject's nutritional status.

Independent variable	*t*	*B *	*P*
BMI Model (*R* ^2^ = 0.190, *F* = 8.451, *P* < 0.001)			
PEF (L/s)	2.450	0.644	0.001
Plasma vitamin A (*μ*mol/L)	2.845	1.856	0.005
Handgrip (kg)	2.616	0.197	0.010

FFMI Model (*R* ^2^ = 0.081, *F* = 5.309, *P* = 0.007)			
FEV_1_ (L)	2.436	1.297	0.017
Plasma vitamin A (*μ*mol/L)	2.152	0.948	0.034

*P* < 0.05 significant using multiple linear regression analysis

Adjusted for BMI: income, handgrip strength, vitamin E intake, FEV_1_% predicted, FEV_1_, PEF, PEF% predicted, and plasma vitamin A.

Adjusted for FFMI: handgrip strength, FEV_1_% predicted, FEV_1_, PEF, PEF% predicted, and plasma vitamin A.
